# Inflammatory bowel disease in paediatric rheumatological diseases

**DOI:** 10.1007/s10067-025-07424-w

**Published:** 2025-04-04

**Authors:** Özen Taş, Fatma Aydın, Zarife Kuloğlu, Ceyda Tuna Kırsaçlıoğlu, Onur Bahçeci, Betül Öksüz Aydın, Doğacan Sarısoy, Zeynep Birsin Özçakar

**Affiliations:** 1 Division of Pediatric Rheumatology, Department of Pediatrics, Ankara University School of Medicine, Ankara, Turkey; 2 Division of Gastroenterology, Hepatology and Nutrition, Department of Pediatrics, Ankara University School of Medicine, Ankara, Turkey; 3 Division of Pediatric Nephrology and Rheumatology, Department of Pediatrics, Ankara University School of Medicine, Ankara, Turkey

**Keywords:** Chronic nonbacterial osteomyelitis, Familial Mediterranean fever, Inflammatory bowel disease, Juvenile idiopathic arthritis, Rheumatological diseases

## Abstract

**Introduction:**

Rheumatological diseases (RD) in childhood are systemic diseases that occur on the basis of auto-immunity or inflammation, and they can be accompanied by inflammatory bowel disease (IBD). When there is no knowledge of this association, the treatments applied may not be sufficient and/or treatments given for RD may even lead to aggrevation of IBD findings. Thus, early identification of an association is crucial for the correct management of the diseases.

**Objectives:**

The aim of this study is to show the frequency of IBD in patients with RDs. We also aimed to investigate in which cases IBD should be suspected in children with RDs.

**Methods:**

Electronic medical records of the patients who were followed up between 2012 and 2024 with a diagnosis of RD in our Paediatric Rheumatology Unit and diagnosed with IBD were reviewed retrospectively.

**Results:**

Between 2012 and 2024, 20 (3%) of 650 familial Mediterranean fever (FMF) patients, 3 (7.5%) of 40 chronic nonbacterial osteomyelitis (CNO) patients (one of them also had FMF) and 2 (1.2%) of 170 juvenile idiopathic arthritis (JIA) patients were diagnosed with IBD. While 15 (62.5%) of the patients received a RD as the initial diagnosis, 9 (37.5%) of them were initially diagnosed with IBD and then referred to rheumatology for their symptoms.

The median age at the diagnosis of RD was 9 years (inter quartile range (IQR), 14.5). The median age at the diagnosis of IBD was 12 years (IQR, 13), and 12 patients (50%) had Crohn’s disease (CD), 10 patients (41.6%) ulcerative colitis (UC) and 2 patients (8.4%) undeterminated disease. Although majority of the patients had classical findings of IBD, 4 patients presented with more vague symptoms including treatment-resistant iron deficiency anaemia, perianal abscess, weight loss and growth retardation.

**Conclusions:**

RD and IBD share similar pathological pathways and clinical findings, and IBD can accompany to various RD. The diagnosis of IBD should be considered in the presence of rare and atypical symptoms. Furthermore, RD should also be considered in children with IBD who have complex extraintestinal symptoms.

**Table:** 

**Key Points** • *RD, especially FMF, JIA and CNO, may be associated with IBD*. • *Paediatric rheumatologists should consider IBD as a potential diagnosis in the presence of atypical findings that may develop during the course of RD*. • *The co-existence of RD and IBD is important both in terms of disease progression and treatment decisions*.

## Background and objectives

Rheumatological diseases (RD) in childhood are systemic diseases that occur on the basis of autoimmunity or inflammation and affect various organs, including joints, skin, muscles, vessels, bones, eyes, lungs and intestines [1]. Immune dysregulation involving innate or acquired immunity is postulated to play a role in RD. In this context, diseases that develop as a result of immune dysregulation are expected to occur together. Among these diseases, familial Mediterranean fever (FMF), juvenile idiopathic arthritis (JIA) and chronic nonbacterial osteomyelitis (CNO) may be associated with other inflammatory diseases such as inflammatory bowel disease (IBD), and the course of these diseases may change [2].

IBD is a chronic inflammatory disease of the gastrointestinal tract with highly variable clinical signs and symptoms. Although the cause is not fully explained, there are some studies that draw attention to the coexistence of IBD and RD [3, 4].

FMF is the most common autosomal recessively inherited monogenic autoinflammatory disease, and IBD is the most common form of chronic intestinal inflammation. IBD and FMF are both autoinflammatory diseases that present with concomitant symptoms such as abdominal pain, diarrhoea, fever and arthralgia/arthritis. FMF is distinguished from IBD by recurrent attacks of symptoms, the presence of neutrophilic infiltration in the serosal tissues and dysregulation of apoptosis [5, 6]. In FMF, mutations in the MEFV gene trigger inflammation affecting many tissues through secretion of interleukin- 1beta (IL- 1b) and activation of the NF-kappa B pathway. Therefore, FMF is more commonly associated with other inflammatory diseases such as IBD, especially in regions where FMF is endemic. The high frequency of association may be attributed to shared epigenetic factors, the coexistence of independent founder effects, or shared genetic factors [7].

CNO is a rare autoinflammatory bone disease affecting children and adolescents. CNO and IBD result from an abnormal immune response with the release of pro-inflammatory cytokines and other immune mediators contributing to the observed inflammation [8]. This is thought to involve dysregulation of IL- 10, as polymorphisms in its promoter region have been observed in IBD patients [9]. In addition, intestinal immune responses can affect joints, with TNF-α, IL- 12 and IL- 23 playing a crucial role. TNF-α upregulation has been observed in IBD, JIA and CNO, explaining the positive response to anti-TNF-α therapy in these diseases [10]. IL- 12 has been identified as a marker of treatment response in CNO patients [11]. The involvement of common cytokines and immune cells suggests a shared immunological basis. Genetic markers and susceptibility genes have been identified for both conditions, suggesting the presence of common genetic factors. Mutations in the NLRP3 gene, associated with autoinflammatory syndromes, have been identified in IBD patients and some CNO patients. In addition, a limited group of patients with CNO have been tested for known susceptibility genes associated with IBD, such as the CARD15/NOD2 gene [12, 13].

JIA is the most common chronic inflammatory disease in children. TNF-α, a pro-inflammatory cytokine, plays a central role in the pathophysiology of inflammation. TNF-α secretion has also been implicated in the pathogenesis of both IBD and JIA [14].

The treatment of RD, especially when there is a comorbidity such as IBD, may get difficult. Paediatric patients whose growth and development continue can be adversely affected. In some cases, treatment of RD may even lead to the exacerbation of the symptoms of IBD [15]. Therefore, early recognition of the association between RD and IBD is very important for effective management of the disease course and prevention of long-term complications. However, the diagnosis of IBD can be difficult in patients with occult or overlapping findings.

The aim of this study is to demonstrate the frequency of coexisting RD and IBD and to investigate when IBD should be suspected in children with RD.

## Patients and methods

The patients diagnosed with both IBD and RD in our paediatric rheumatology unit between 2012 and 2024 were recruited to the study. The electronic medical records of the patients were retrospectively reviewed for demographic features, clinical findings, suspicious symptoms for requiring referral to the paediatric gastroenterology department (PGD) during rheumatology clinic follow-up or vice-versa, laboratory, endoscopic and histopathologic findings. Among the patients followed up for rheumatic diseases in the paediatric rheumatology clinic, those with a concomitant diagnosis of IBD were rewieved. These patients were compared with patients followed up in the gastroenterology clinic with a diagnosis of IBD and also with a diagnosis of rheumatological disease to avoid missing patients and missing data. Therefore, in this study, we included all patients in the intersection cluster of patients with a diagnosis of IBD and rheumatological diseases.

In diagnosing FMF, the Yalçınkaya criteria were used [2]. There are no universal diagnostic criteria for CNO, but the widely used Jansson criteria and Bristol criteria rely on a combination of clinical and imaging findings [16, 17]. After ruling out infectious and malignant causes, patients were diagnosed with CNO based on clinical and magnetic resonance imaging (MRI) findings. Patients who met the International League of Associations for Rheumatology (ILAR) criteria for JIA were included in the study. The ILAR criteria classify JIA into several subtypes based on specific clinical features and disease duration as systemic arthritis, oligoarthritis, polyarthritis, enthesitis-related arthritis, psoriatic arthritis and undifferentiated arthritis [18].

The patients who had suspicious clinical and/or labarotory findings of IBD were evaluated by upper gastrointestinal endoscopy (UGIE) and ileocolonoscopy by an experienced paediatric gastroenterologist under deep sedation. Multiple biopsies were taken from each part of gastrointestinal tract for histopathological examination. In respect of clinical, laboratory, endoscopic and histopathological findings IBD diagnosed according to European Society of Pediatric Gastroenterology, Hepatology, and Nutrition (ESPGHAN) revised Porto criteria and paediatric IBD subtypes Crohn’s disease (CD), ulcerative colitis (UC) and IBD-undetermined (IBD-U) were defined according to ‘validated paediatric IBD-classes algorithm’ [19, 20]. The severity of IBD was categorised as mild, moderate and severe by the paediatric CD activity index and paediatric UC activity index [21, 22]. Because of a lack of specific criteria, disease activity and involvement in the children with IBD-U were assessed as in UC [22].

It was recorded whether the initial diagnosis was RD or IBD, or whether it was concurrent.

The study was approved by local ethics committee (number/date: 2,024,000,269–1/18.04.2024) in accordance with ethical principles outlined in the Declaration of Helsinki.

### Statistical analysis

Descriptive statistics were presented as mean ± standard deviation for the variables distributed normally (Shapiro–Wilk test) and as median [inter quartile range (IQR)] for the variables distributed not normally. In contrast, they were presented as numbers and percentages (%) for nominal variables. The analyses were conducted using the Statistical Package for Social Sciences (SPSS, Version 22.0, Chicago, IL).

## Results

Twenty four patients with RD [3% of patients with FMF (20/650 patients) (2 *patient had concurrent sacroiliitis)*, 7.5% of patients with CNO (3/40 patients) (*1 patient had concurrent FMF)*, and (1.2%) of patients with JIA (2/170 patients)] were diagnosed with IBD. Seventeen patients (68%) had only FMF, 2 (8.4%) had FMF and sacroiliitis, 1 (4.2%) had FMF and CNO, 2 (8.4%) had CNO and 2 (8.4%) had JIA. The median age of the patients was 15 years (IQR, 19). Fifteen patients (62.5%) were initially diagnosed with RD, and 9 patients (37.5%) were initially diagnosed with IBD. Of the 24 patients, 12 (50%) had CD, 10 (41,6%) had UC and 2 (8,4%) had IBD-U. Demographic and clinical data of the patients are shown in Table 1.

**Table 1:** The demographic, clinical and genetic features of participants

	*n* = 24 (%) Median (IQR)
Female	8 (33)
Age (years)	15 (19)
Age at diagnosis (years)	
Rheumatological disease	9 (14.5)
Inflammatory bowel disease	12 (13)
First diagnosis	
Rheumatological disease	15 (62.5)
Inflammatory bowel disease	9 (37.5)
Rheumatological disease	
FMF	16 (66.6)
FMF + sacroiliitis	2 (8.4)
FMF + CNO	1 (4.2)
FMF + vasculitis	1 (4.2)
CNO	2 (8.4)
JIA	2 (8.4)
Clinical findings related to rheumatological disease	
Abdominal pain	15 (62.5)
Fever	14 (58.3)
Arthritis/Arthralgia	13 (54.2)
Lower back pain	3 (12.5)
Heel pain	3 (12.5)
Chest pain	2 (8.3)
Leg pain	2 (8.3)
Morning stiffness	2 (8.3)
Oral aphthae	1 (4.2)
Amyloidosis	1 (4.2)
Growth retardation	1 (4.2)
Inflammatory bowel disease	
Crohn’s disease	12 (50)
Ulcerative colitis	10 (41.6)
Inflammatory bowel disease-undetermined	2 (8.4)
Clinical findings related to inflammatory bowel disease	
Chronic/recurrent diarrhea	19 (79.2)
Recurrent abdominal pain	11 (45.8)
Weight loss	5 (20.8)
Fever	4 (16.7)
Anal lesion	3 (12.5)
Iron deficiency	3 (12.5)
Erythema nodosum	2 (8.3)
Oral aphthae	2 (8.3)
Growth retardation	1 (4.2)
MEFV mutations of patients	
M694 V/M694 V	5 (25)
Biallelic exon 10 mutations *(other than M694 V/M694 V)*	7 (35)
Monoallelic exon 10 mutations	2 (10)
Other mutations	6 (30)

*IQR* inter quantile range

*CNO* chronic nonbacterial osteomyelitis

*FMF* familial Mediterranean fever

*JIA* juvenile idiopathic arthritis

*MEFV* Mediterranean fever

The most common clinical findings of rheumatological diseases were abdominal pain (62.5%), fever (58.3%) and arthritis/arthralgia (54.2%). The most common clinical findings regarding to IBD were chronic diarrhoea (79.2%) and chronic abdominal pain (45.8%).

The most commonly observed suspicious symptoms in patients initially diagnosed with rheumatological conditions and then subsequently referred to the gastroenterology department were recurrent/chronic diarrhoea in 9 (60%), recurrent abdominal pain in 6 (40%), bloody stools in 4 (26.6%) and unexplained elevated acute phase reactants (APR) in 4 patients (26.6%). Other symptoms that prompted referral to the gastroenterology department included treatment-resistant iron deficiency (20%), erythema nodosum (20%), weight loss (13.3%), vitamin B12 deficiency (6.6%), growth retardation (6.6%), anal lesion (6.6%) and fever (6.6%).

The most commonly observed suspicious symptoms in patients initially diagnosed with IBD and then subsequently referred to the rheumatology department were fever in 5 (55.5%) and abdominal pain incompatible with the initial diagnosis in 4 patients (44.4%). Other symptoms that prompted referral to the rheumatology department included elevated APR without an identifiable cause (22.2%), lower back pain (22.2%), arthralgia (22.2%), heel pain (11.1%), leg pain (11.1%), recurrent oral aphthae (11.1%) and proteinuria (11.1%).

Of the patients, 45.8% (11 patients) had early-onset IBD (< 10 years old age) and 81.8% (9 patients) of them had very early onset IBD (< 6 years old age). The severity of IBD at diagnosis was as follows: 75% had mild disease, 12.5% had moderate disease and 12.5% had severe disease. Of the patients, 70.8% (12 UC, 4 CD, 1 IBD-Undetermined colitis (IBD-UC) had only colonic, 25% (6 patients; 6 CD) had ileoconic and 4.7% (1 patient with CD)) had only ileal involvement. The perianal disease was observed in 12.5% of the 24 patients.

The detailed demographic and clinical characteristics of all patients who were first diagnosed with RD and subsequently diagnosed with IBD and who were first diagnosed as IBD and subsequently diagnosed with RD are presented in Tables 2 and 3, respectively. The treatments of the patients were given in Table 4.

**Table 2:** Baseline characteristics and clinical findings of patients with an initial rheumatological diagnosis

Patient #	Gender	Presenting age/symptoms	Initial diagnosis age/RD	Age/IBD-related symptoms	IBD diagnosis age/IBD type (severity/location)	Follow-up duration (years)
*Patient 1*	M	8 years/morning stiffness, hip pain and limping	9 years/JIA	15 years/weight loss, iron deficiency anaemia, elevated APR	15 years/UC (mild/colon)	8
*Patient 2*	M	8 years/arthritis in the ankle, hip and heel pain	9 years/JIA	11 years/erythema nodosum, RAP, recurrent diarrhea, perianal abcess	15 years/CD (mild/ileocolon)	7
*Patient 3*	M	1 year/RAP, RF	2 years/FMF	8 years/RAP, chronic diarrhea, intermittently blood (+)	8 years/Atypical UC (mild/colon)	16
*Patient 4*	F	2.5 years/RAP, RF	7 years/FMF	15 years/iron deficiency anaemia	18 years/CD (mild/ileum)	22.5
*Patient 5*	M	3.5 years/RAP, RF, chest pain	4 years/FMF	3.5 years/RAP, fever, haematochezia, weight loss, erythema nodosum	7 years/UC (mild/colon)	8.5
*Patient 6*	F	2 years/RAP, RF	4 years/FMF	4 years/RAP, diarrhea	5 years/Atypical UC (mild/colon)	12
*Patient 7*	M	4.5 years/RAP, RF knee pain, chest pain	6.5 years/FMF	11 years/chronic diarrhea	13 years/CD (mild/ileocolon)	10.5
*Patient 8*	M	6 years/RAP, RF low back pain, heel pain and chest pain	12 years/FMF	12 years/growth retardation, iron deficiency anaemia, intermittent diarrhea	13 years/CD (mild/ileocolon)	13
*Patient 9*	F	14 years/RAP, RF	16.6 years/FMF	14 years/RAP, recurrent diarrhea	18 years/UC (mild/colon)	9
*Patient 10*	F	8 years/RAP, RF	8 years/FMF	12 years/RAP, mucoid bloody diarrhea, fever	12 years/UC mild/colon)	13
*Patient 11*	M	9 years/pain in the knee and the ankle, morning stiffness and staggering gait	13 years/CNO	15 years/anal abscess	16 years/CD (mild/colon)	7
*Patient 12*	M	5 years/abdominal pain, fever, arthritis, HSP	6 years/FMF	6 years/weight loss, abdominal pain, bloody diarrhea	6 years/CD (mild/colon)	4
*Patient 13*	M	6 years/arthralgia, arthritis and difficulty in walking	10 years/FMF, sacroileitis	10 years/diarrhea	12 years/CD (mild/ileocolon)	6
*Patient 14*	F	9 years/nonpalpable purpura, RAP, arthritis	9 years/FMF, leukocytoclastic vasculitis	10 years/wasting, chronic diarrhea	10 years/UC (mild/colon)	12
*Patient 15*	F	6 years/RAP, arthralgia	9 years/FMF	14 years/chronic diarrhea	14 years/CD (mild/ileocolon)	19

The severity of IBD was assessed using the PUCAI and PCDAI scores [11, 12]

*F* female

*M* male

*IBD* inflammatory bowel disease

*FMF* familial Mediterranean fever

*CNO* chronic nonbacterial osteomyelitis

*JIA* juvenile idiopathic arthritis

*CD* Crohn’s disease

*UC* ulcerative colitis

*RAP *recurrent abdominal pain

*RD* rheumatological disease

*RF *recurrent fever

**Table 3:** Clinical findings of patients with an initial IBD diagnosis

Patient #	Gender	Presentation age/symptoms	Initial diagnosis age/IBD type (severity/location)	Age/RD related symptoms	RD diagnosis age/RD	Follow up period (years)
*Patient 1*	M	6 months/bloody diarrhea	1 year/CD (severe/colon)	6 months/recurrent fever, abdominal pain, and restlessness	1 year/FMF	16.5
*Patient 2*	F	11 years/abdominal pain, fever and intermittant diarrhea	11 years/IBD-UD (moderate/colon)	15 years/proteinuria (renal amyloidosis)	15 years/FMF	13
*Patient 3*	M	1 month/chronic diarrhea 2.5 years/oral aphthaous ulcers, bloody diarrhea, abdominal pain and fever	3 years/UC (mild/colon)	2 years/recurrent abdominal pain, fever, and rash	3 years/FMF, sacroiliitis	11
*Patient 4*	M	6 months/bloody watery diarrhea, weight loss and growth retardation	1.5 years/IBD-UD (severe/colon)	3 years/lower back pain	3 years/FMF, sacroiliitis	12.5
*Patient 5*	M	7.5 years/abdominal pain, vomiting, bloody diarrhea	10 years/atypical UC (mild/colon)	10.5 years/lower back, heel, hip, knee and ankle pain	12 years/CNO	4.5
*Patient 6*	M	3 years/perianal abcess recurrent aphthaous ulcers, poor weight gain, constipation, and chronic iron deficiency anaemia	12 years/CD (mild/ileocolon)	4 years/recurrent fever, recurrent aphthaous ulcers	13 years/FMF	12
*Patient 7*	M	2.5 years/chronic diarrhea	2.5 years/CD (severe/colon)	3 years/leg pain, fever	4 years/FMF	3.5
*Patient 8*	F	6 months/bloody mucoid diarrhea	2 years/UC (moderate/colon)	1 year/growth retardation	2 years/FMF	6.5
*Patient 9*	M	6 months/weight loss, chronic diarrhea and perianal deep fissures	1.5 years/CD (severe/colon)	6 months/recurrent fever, arthritis	1.5 years/FMF, CNO	10.5

The severity of IBD was assessed using the PUCAI and PCDAI scores [11, 12]

*F* Female

*M* Male

*IBD* Inflammatory bowel disease

*FMF* Familial Mediterranean fever

*CNO* Chronic nonbacterial osteomyelitis

*JIA* Juvenile idiopathic arthritis

*CD* Crohn’s disease

*RD* Rheumatological disease

*UC* Ulcerative colitis

*IBD-UD* IBD-Undetermined disease

**Table 4:** Treatment options used in patients

		Treatment of patients with an initial rheumatological disease diagnosis	
*Patient #*	Diagnosis of rheumatological disease	Treatment for rheumatological disease	Treatment for IBD
*Patient 1*	JIA	NSAID, sulfasalazine, MTX, ADA	ADA
*Patient 2*	JIA	NSAID, sulfasalazine, ADA, infliximab	ADA, infliximab
*Patient 3*	FMF	Colchicine	Mesalazine
*Patient 4*	FMF	Colchicine	Budesonide, AZA
*Patient 5*	FMF	Colchicine	Budesonide
*Patient 6*	FMF	Colchicine	Mesalazine
*Patient 7*	FMF	Colchicine, anakinra, canakinumab	Mesalazine
*Patient 8*	FMF	Colchicine	Budesonide
*Patient 9*	FMF	Colchicine	NR
*Patient 10*	FMF	Colchicine	CS, mesalazine, AZA
*Patient 11*	CNO	NSAID, MTX, infliximab	Mesalazine, infliximab
*Patient 12*	FMF	Colchicine	Mesalazine, AZA
*Patient 13*	FMF+sacroiliitis	Colchicine, NSAID	Mesalazine
*Patient 14*	FMF	Colchicine	Mesalazine
*Patient 15*	FMF	Colchicine	Mesalazine
		Treatment of patients with an initial diagnosis of IBD	
		Treatment for rheumatological disease	Treatment for IBD
*Patient 1*	FMF	Colchicine	Mesalazine, AZA
*Patient 2*	FMF	Colchicine, anakinra	Mesalazine
*Patient 3*	FMF	Colchicine	Mesalazine
*Patient 4*	FMF+sacroiliitis	Colchicine, sulfasalazine, ADA	Mesalazine, CS
*Patient 5*	CNO	NSAID, ADA	ADA, mesalazine
*Patient 6*	FMF	Colchicine	ADA, infliximab
*Patient 7*	FMF	Colchicine	CS, AZA, ADA
*Patient 8*	FMF	Colchicine	Mesalazine
*Patient 9*	FMF+CNO	Colchicine, anakinra, CS, ADA, NSAID	CS, mesalazine, AZA

*FMF* Familial Mediterranean fever, *JIA* Juvenile idiopathic arthritis, *CNO* Chronic non-bacterial osteomyelitis, *IBD* Inflammatory bowel disease, *NSAID* nonsteroidal antiinflammatory drug, *MTX* Methotrexate, *ADA* Adalimumab, *AZA* azathiopurine, *CS* corticosteroid, *NR* not reported

### Presentation of atypical cases

Here, we presented four patients with RD who had atypical symptoms on follow-up.

Case one (Patient 4 in Table 1) is a 7-year-old girl admitted to rheumatology clinic with the complaints of recurrent fever episodes that started at 3 months of age and abdominal pain attacks (every 10 days) starting at 2 and 5 years of age. Colchicine was initiated with the diagnosis of FMF, and she had *M694 V/V726 A* mutation. At 15 years of age, iron deficiency anaemia (IDA) was diagnosed and appropriate iron treatment was arranged. Due to the persistence of IDA despite an appropriate dose and regular use of iron therapy for 15 months, she was referred to the PGD. Ileocolonoscopy revealed ileal aphtous ulceration, and a diagnosis of CD was made following pathological findings of ‘active ileitis’ characterised by erosion and regeneration.

Case two (Patient 11 in Table 1) is a 10-year-old boy, presented with morning stiffness and bone pain for a period of 1 year. Physical examination revealed localised tenderness in the sacroiliac joints, both knees and the thoracic vertebrae. The whole body MRI (WB-MRI) scan revealed multifocal CNO findings at the age of 13, and non-steroid anti-inflammatory drug (NSAID) and methotrexate (MTX) treatments were commenced. He presented with recurrent anal abscess (2 times with an interval of 1 year) in the second year of the diagnosis of CNO, and then he was referred to the PGD. The UGIE revealed pangastritis, bile reflux and nodularity on the second part of the duodenum. Ileocolonoscopy revealed normal terminal ileum mucosa but hyperemia, oedema, fragility and loss of vascularity throughout the entire colon. Histopathological examination revealed diffuse active colitis, characterised by erosion, cryptitis, crypt abscess and crypt distortion throughout the colon mucosa. With the diagnosis of colonic CD, infliximab treatment was initiated, and he is currently being monitored in remission with these treatments.

Case three (Patient 1 in Table 1) is a 9-year-old boy presented with the complaints of pain in the ankles, heel, knee and hip. MRI of the patient revealed active sacroiliitis, and the patient was diagnosed with enthesitis-related arthritis (ERA). NSAID and salazoprine treatments were initiated. Furthermore, methotrexate treatment was administered for a period of approximately 2 years during the course of the patient’s illness. He had weight loss, IDA and elevated APRs (CRP (C-reactive protein), 68.1 mg/dL; erythrocyte sedimentation rate (ESR), 51 mm/h); and faecal calprotectin (702 mcg/g, reference value < 80 mcg/g) levels, and referred to the paediatric gastroenterology department at the age of 15 years. The UGIE revealed a 3 × 3 mm superficial ulcer on the duodenal bulb mucosa; ileocolonoscopy revealed hyperemia, fragility, friability and diffuse ulcerations extending from the rectum to the cecum, ulcerations on the ileocecal valve and terminal ileum mucosa. Histopathology revealed normal duodenal and terminal ileum mucosa, but fovealar metaplasia and cryptitis on duodenal bulb mucosa, diffuse active colitis with mild cryptitis and crypt distorsion throughout the colon. He was diagnosed as UC. At present, both rheumatological findings and IBD symptoms are being effectively managed through the implementation of adalimumab treatment.

Last case (Patient 8 in Table 1), now 19 years of age, first exhibited symptoms at the age of six with recurrent fever accompanied by abdominal pain, heel pain and high APRs at hospital admissions. Upon presentation to our clinic at the age of 12, we identified a homozygous *M694 V* mutation and was diagnosed with FMF. Colchicine treatment was initiated. During follow-up visits that year, growth retardation (height < 3p and weight < 3p) was recognised, and he was referred to PGD. The UGIE revealed an oedematous appearance at the lower end of the oesophagus, while the pathology revealed oesophagitis characterised by congestion and papullomatous lesions. Ileocolonoscopy revealed aphtous ulcers on terminal ileum and normal colon mucosa. Severe active ileitis and mild active colitis were observed on histopathological examination and a diagnosis of CD was made.

## Discussion

IBD can be seen in patients with RD. In this study, we found that IBD may coexist with FMF, CNO and JIA during childhood period. Although majority of IBD patients had classical clinical findings, approximately 20% had occult findings requiring sceptical approach.

The pathogenesis of IBD is thought to be multifactorial, involving genetic predisposition, immune dysregulation, environment and microbiome. Paediatric IBD can present with abdominal pain, chronic bloody or watery diarrhoea, weight loss, anorexia, growth delay, delayed puberty, perianal disease and/or extraintestinal symptoms such as arthritis, apthous ulceration and erythema nodosum [23]. Given the shared pathogenetic mechanisms between RD and IBD, it is possible that patients with RD may develop IBD at any stage of disease. Besides that, rheumatological manifestations are commonly encountered in individuals with IBD, with peripheral arthritis, axial involvement, peripheral enthesitis and secondary osteoporosis being particularly prevalent [24]. In a study conducted by Repiso and colleagues, 46% of 157 patients with CD exhibited extraintestinal manifestations, while 22% demonstrated rheumatological findings [25]. Furthermore, the presence of shared clinical findings can also contribute to confusion. For instance, joint findings may develop as extraintestinal manifestations of IBD, or they may also be signs of accompanying rheumatological diseases such as JIA, CNO and FMF. On the other hand, while abdominal pain can be a symptom of an attack of FMF, the chronic diarrhoea that accompanies can be a warning sign. In these patients, the presence of other associated clinical symptoms and, on occasion, imaging results can provide valuable guidance.

FMF is the most common inherited autoinflammatory disease, and it is a disease that causes inflammation. Vasculitis, sacroiliitis and IBD may be associated with FMF as they all develop on the basis of inflammation [26, 27]. Previous studies have demonstrated that IBD is prevalent in patients with FMF [28]. Cattan et al. demonstrated that the association between FMF and IBD in non-Ashkenazi Jews was 8–14 times greater than expected in adult patients [28]. Mutations in the *MEFV (Mediterrenean FeVer)* gene, which encodes the pyrin protein, lead to activation of the pyrin inflammasome, overproduction of certain inflammatory cytokines, particularly IL- 1, and uncontrolled pyroptotic cell death. Pyroptosis is a form of gasdermin D (GsdmD)-mediated inflammatory cell death involving inflammatory caspases [29]. GsdmD also plays an essential role in the pathogenesis of IBD [30]. In the absence of treatment, the progression of these diseases may result in the unchecked activation of GsdmD-mediated pyroptotic cell death, which can lead to permanent damage to the intestinal mucosa [31]. Moreover, the absence of autoantibodies and antigen-specific T-cells in autoinflammatory diseases suggests that dysregulation of the innate immune system plays a role in disease development. The *MEFV* gene and the CD susceptibility gene NOD2/CARD15 (nucleotide-binding oligomerization domain-containing protein 2/caspase recruitment domain-containing protein 15) are located on chromosome 16 [32]. The *MEFV* gene encodes pyrin (marenostrin) and NOD2/CARD15 produces death domain proteins. These two genes are involved in the same common signalling pathway, encoding proteins that interact with inflammasomes and activate caspase- 1. In a study conducted by Ataş and colleagues, 971 patients with FMF were analysed. The findings revealed that 14 patients (1%) had an underlying IBD concurrently present [33]. In a separate study conducted by Yıldız et al. in paediatric patients with FMF, it was observed that 10 (1.5%) of the 686 FMF patients had IBD [34]. In our study on the other hand, 20 out of 650 FMF patients (3%) were diagnosed with IBD.

JIA is a common childhood RD with chronic arthritis [35]. ERA is one of the subgroups of JIA and may be seen especially in association with IBD [36]. The IL- 23/IL- 17 pathway is believed to be a significant factor in the pathogenesis, particularly in conjunction with HLA (human leukocyte antigen)-B27 positivity [37]. IL- 17 A and IL- 17 F are a pair of recently identified pro-inflammatory cytokines that can bridge the gap between the adaptive and innate immune systems. Both cytokines are involved in the maintenance of epithelial barrier function and protection against pathogens [38]. However, alterations in the regulation and excess of these two cytokines have pathogenetic implications in chronic immune-mediated inflammatory diseases, including psoriasis (PsO), psoriatic arthritis (PsA), axial spondyloarthritis (axSpA), hidradenitis suppurative (HS) and IBD. A number of different cell types and studies have highlighted the potential importance of bacterial dysbiosis, but the precise nature of the relationship between this phenomenon and the development of IBD remains unclear. A study utilising data from the German Biologics registry (Biologika in der Kinderrheumatologie; BiKeR) encompassed 3071 JIA patients with 8389 patient-years (PY) of observation. IBD was diagnosed in 11 patients (0.4%), comprising 8 with CD and 3 with UC [39]. A total of 1651 JIA patients (all were on etanercept treatment) were enrolled in the Dutch, Finnish, Danish, German and Italian registries between 1999 and 2008. The Dutch registry enrolled 226, the Finnish registry 150, the Danish registry 146, the German registry 1006 and the Italian registry 203 patients. A total of 17 (1.02%) patients were diagnosed with IBD in these national JIA registries (three Dutch cases, eight German cases, five Italian cases and one Finnish case) [40–43]. In our study, IBD was observed in 2 out of 170 (1.2%) JIA patients.

CNO is a disease characterised by auto-inflammation of bones and has also been reported in association with IBD. Three main pathophysiological mechanisms have been hypothesised to drive CNO, including inappropriate cytokine expression, increased inflammasome activation and enhanced osteoclast differentiation. A genetic predisposition has been proposed, with familial clustering and a strong association with inflammatory disorders of the skin and intestinal tract in affected individuals and among close relatives. It has been reported that families of patients with CNO have a higher prevalence of inflammatory conditions such as CD, UC, celiac disease and ankylosing spondylitis [44]. Although the exact processes underlying the development of IBD and CNO are not fully understood, several possible theories have been proposed. They share common pathophysiological mechanisms, including increased pro-inflammatory cytokines and dysregulation of IL- 10 [9]. The microbiome is another common mechanism in the development of both IBD and CNO [45]. Common genetic factors, such as genetic markers and susceptibility genes, have been identified for both conditions [46]. Up to 20% of patients with CNO have extra-bone manifestations involving the skin (psoriasis, palmoplantar pustulosis) or IBD (UC or CD) [47]. The exact prevalence of the coexistence has not been documented yet. In a study which was a retrospective chart review, conducted in 2021, a total of 600 paediatric patients diagnosed with IBD and 47 with CNO were included. Seven out of 47 patients with CNO (14.8%) had IBD. On the other hand, 7 out of 600 IBD patients (1.16%) were diagnosed with CNO [48]. In our study, 3 of 40 CNO patients (7.5%) were diagnosed with IBD. Furthermore, in a review, 40 patients with both CNO and IBD were evaluated. Among these patients, 21 (52.5%) were initially diagnosed with CNO, while 14 (35%) were initially diagnosed with IBD. Five patients (12.5%) were diagnosed simultaneously [46].

In some cases, the drugs used may be a trigger for IBD. It has been reported that some JIA patients have developed IBD while receiving etanercept [40]. However, neither of our JIA patients who developed IBD were taking etanercept. The patients’ treatments were arranged considering both diseases.

With this study, we wanted to emphasise that IBD is more easily considered in the presence of typical gastrointestinal symptoms such as abdominal pain associated with bowel movements, tenesmus, lack of appetite and diarrhoea with or without blood in patients with rheumatological diseases, whereas IBD should be considered in the presence of atypical symptoms and in cases where inflammation persists while the primary disease is under control. It is imperative to reiterate the following point; cases of unexplained fever, arthritis or arthralgia, back pain, and rash in IBD patients should be considered, irrespective of the presence of a rheumatological diseases.

The limitations of this study are its retrospective nature, its reliance on medical records, the small number of patients and the lack of a control group. Another limitation was the inability to perform advanced statistical analysis due to the small number of patients.

In conclusion, RD, especially FMF, JIA and CNO, may be associated with IBD, usually with typical and sometimes atypical manifestations. The diagnosis of IBD could be established either before or after the diagnosis of RD or simutaneously as in our study. In light of the above information, it is imperative to emphasise the need to consider IBD as a potential diagnosis in the presence of atypical findings that may develop during the course of RD. This is of paramount importance in terms of treatment choice and efficacy. The co-existence of two diseases is important both in terms of disease progression and treatment decisions. Treatment options should be chosen that cover both conditions and do not make one condition worse while improving the other.

## Data Availability

Data are available to the public when a reasonable request is made.
